# Associations between remote patient monitoring and uncontrolled blood pressure among patients diagnosed with hypertension: Exploring variations by race/ethnicity

**DOI:** 10.1371/journal.pone.0334887

**Published:** 2025-11-06

**Authors:** John M. Meddar, Devin Mann, Mark Schwartz, Hyung G. Park, Rachel Engelberg, Maria R. Khan

**Affiliations:** 1 Department of Population Health, New York University Grossman School of Medicine, New York, New York, United States of America; 2 Division of General Internal Medicine, Department of Medicine, Mount Sinai Hospital, New York, New York, United States of America; Northwell Health Feinstein Institutes for Medical Research, UNITED STATES OF AMERICA

## Abstract

**Background:**

Hypertension (HTN) is a critical public health concern that disproportionately impacts racial/ethnic minorities. The recent COVID-19 pandemic spurred rapid adoption of virtual HTN treatment programs such as remote patient monitoring programs (RPM), including among minority populations. However, it is unclear how utilization patterns differ across racial/ethnic groups and what the implications are for HTN outcomes.

**Objective:**

The present study examines whether the association between RPM utilization and uncontrolled BP differs by race/ethnicity among hypertensive patients enrolled in an RPM program.

**Methods:**

This study includes an urban sample of HTN patients who were 18 ≥ years old who have been in their RPM programs for three consecutive months or longer. Our primary exposure measures are three widely used dichotomized RPM engagement metrics and uncontrolled BP outcomes were dichotomized as BP ≥ 140/90 and ≥ 130/80. We tested for effect modification by race/ethnicity across RPM utilization variables using multivariable logistic regression models.

**Results:**

Of 2920 participants, 59% were females, 37% were ≥ 65 years old, and Hispanic patients were the most represented race/ethnicity group (39%). Percentage-uncontrolled was 25% non-Hispanic Black, 21% Hispanic, and 20% among non-Hispanic White patients. Compared to non-Hispanic White patients with high RPM utilization, patients with no BP transmission had higher odds of uncontrolled BP: White (OR=1.72; 95% CI: 1.07–2.75), Black (OR=2.11; 95% CI: 1.32–3.39), and Other race (OR=2.36; 95% CI: 1.41–3.96). Similar patterns were observed for low clinician interactions and low portal use.

**Conclusion:**

Disparities in RPM utilization and BP outcomes in our study parallel reported inequities in digital technology utilization and uncontrolled BP in the U.S. Future studies should aim to understand how utilization trends among various vulnerable populations influence HTN outcomes. Such findings may help inform efforts aimed at streamlining access and utilization of RPM to reduce utilization disparities and promote better BP control.

## Introduction

Hypertension (HTN) is a significant public health concern in the United States [1]. Approximately 116 million adult Americans have HTN, and greater than 70% of these have uncontrolled HTN [2,3]. Among these, racial/ethnic minority groups are significantly and disproportionately impacted relative to their non-Hispanic White counterparts [4,5]. While available evidence suggests that HTN control rates, which are linked to increased cardiovascular morbidity and mortality, have decreased in recent years across all racial/ethnic groups, the risk of adverse cardiovascular sequelae is particularly pronounced among racial/ethnic minorities [6,7].

The COVID-19 pandemic triggered the rapid diffusion of telemedicine, which spurred increased participation in HTN telemonitoring programs, including remote patient monitoring (RPM) [8]. HTN- RPM programs use information and telecommunication technologies to remotely monitor and manage diverse aspects of the HTN treatment process [9,10]. RPM has shown significant promise for lowering BP levels and improving clinical outcomes across a range of conditions, including HTN, diabetes, weight management, etc. [11–13].

At New York University Langone Health (NYULH), an urban academic medical center in New York City, RPM programs offer multiple treatment components for HTN management, including remote BP measurement transmission, which are self-measured BP readings transmitted directly to clinicians’ electronic health record (EHR) for real-time monitoring [14]. The program encourages increased and consistent engagement with clinicians through video and telephone visits and asynchronous messaging. It provides an easy-to-use online patient portal, which enables access to lab results, secure patient-clinician communication, electronic bill paying, and other essential components of treatment. In addition, NYULH’s RPM program offers digital modules that support symptom monitoring and provide a range of resources, including educational materials, reminders, alerts, and disease-specific instructions to inspire engaged and proactive self-management practices.

While emerging evidence supports the efficacy and utility of digitally-enabled treatment programs, [12,15,16] various minority populations are excluded from reaping its benefits due to the existing digital divide. For example, diverse populations across rural and urban contexts, including racial/ethnic minorities, rural residents, people with lower levels of income and education, people with disabilities, older adults, and people in tribal lands, are adversely impacted by limited access to quality internet, including broadband connectivity, and digital devices [17–19]. Digital disenfranchisement of these populations inhibits critical engagement with increasingly routinized service delivery technologies, platforms, solutions, and healthcare access points [20–22]. An emerging literature has explicitly reported on the impact of digital inequities on racial/ethnic minorities’ ability to participate in RPM programs. This is further exemplified by the recent expansion of telemedicine, which largely showed increased adoption by non-Hispanic White patients relative to their racial/ethnic minority counterparts [23].

Prior research has examined the frequency of RPM utilization by race/ethnicity across process measures such as BP reading transmissions [24–26]. One study assessed self-measured BP by race/ ethnicity, coupled with adjunctive strategies such as educational initiatives and observed significant reductions in BP outcomes (Systolic BP 19.1 mm Hg/ Diastolic BP 14.8 mm Hg) among non-Hispanic Black and Hispanic men relative to baseline assessments [27]. Another clinical trial examined average reductions in self-measured BP compared with usual care among racial/ethnic minorities and found self-measured BP monitoring to be comparable to usual care (intervention group: 14.7 mm Hg vs control group: 14.1 mm Hg; P = 0.70) [28]. However, no study to our knowledge has explored the explicit associations between race/ethnicity and multiple RPM engagement metrics and BP outcomes.

The primary aim of this exploratory study is to assess whether the strength of the association between RPM utilization and uncontrolled BP differs by racial/ethnic group. We examined race/ethnicity as an effect modifier across three distinct and widely used RPM program utilization indicators: self-measured BP transmission, interactions with clinicians, and engagement with patient portals. To confirm the results, we also assessed the presence of effect measure modification using an alternative threshold for uncontrolled BP.

## Methods

### Participants

In this retrospective cohort study, we queried all hypertensive patients from NYULH’s Epic EHR (Verona, WI) database systems to identify patients who met our inclusion criteria. (**Table 1**) Eligible patients were all hypertensive patients 18 years and older who were currently or had previously been enrolled in an HTN RPM program between January 1, 2018 and January 1, 2024. The study’s baseline was defined as the first in-office BP measurement after enrollment into the program. Patients were identified using a primary diagnosis of HTN in patients’ electronic medical records using ICD-10 codes. Since we aimed to assess the influence of our engagement metrics within the context of a digitally-enabled program, participants were included regardless of utilization history (i.e., participants with high RPM use and no RPM use in the program were likewise included). Participant engagement was assessed over a 3-month study period. Individuals whose RPM episodes were resolved before the 3-month mark were not included. A total of 2,920 participants were deemed eligible for inclusion. Demographic, clinical, and RPM utilization information were extracted from NYULH’s Epic databases for all patients.

**Table 1:** Study inclusion criteria.

Inclusion Criteria
All patients above 18 years old
Had a primary diagnosis of HTN that was recorded by ICD-10 codes
Participant’s RPM episode was not “Resolved” before three months
Had or did not have a history of RPM utilization during the study period

## Measures

### Primary outcomes

We used the Seventh Report of the Joint National Committee (JNC 8) guidelines [29] to define our primary outcome variable. The JNC-8 guidelines prescribe uncontrolled BP as >140 mm Hg systolic BP (SBP) and >90 mm Hg diastolic BP (DBP). We extracted outcome measures after two follow-up periods: the first follow-up period reflects the first 3 months following the 3-month exposure ascertainment period. The second follow-up period corresponds to BP outcome extractions up to 12 months after the first follow-up period, reflecting a combined 15-month outcome ascertainment period. Clinic-based BP values were prioritized and extracted at the first follow-up period or after, if they were available. If clinic-based BP values were not available, we extracted the most recently submitted self-measured BP values by taking an average of the most recent 3 or 2 measurement transmissions, depending on availability. We extracted the averaged values up to 15 months after the exposure ascertainment period. If there were no clinic-based BP values and < 2 BP transmissions, we used the most recently submitted BP measurements, up to 15 months. A 12-month extension period was provided following the 2020 National Quality Forum Measure 0018 (NQF0018) guidelines for BP outcome assessment [30,31]. While this measure uses a 12-month performance period to assess BP outcomes, we provided an additional three months due to the overall lack of BP value submission in our cohort. We dichotomized the extracted values using the above-referenced JNC-8 diagnostic criteria for uncontrolled BP (>140 SBP/ > 90 DBP) as those who met the threshold for uncontrolled BP vs those who did not. Additionally, participants who had no BP measurements within the established 15-month BP outcome assessment period were also classified as uncontrolled (yes vs. no), following the NQF0018 guidelines.

### Secondary outcomes

Our secondary endpoint followed the same analytical procedure, but using the American College of Cardiology and the American Heart Association (ACC/AHA) definition [32] for uncontrolled HTN: SBP > 130 mm Hg/ DBP > 80 mm Hg. Similarly, outcomes were dichotomized based on whether participants met the threshold for uncontrolled BP or not (yes vs. no).

### Primary exposure

BP measurement transmission is defined as any BP transmission vs no BP transmission during the study period. Recording and transmitting BP values involve the use of remote BP cuffs to record and upload BP measurements by manual input or by using Bluetooth-connected RPM devices, which automatically transmit BP values to the clinician’s EHR system. Given very low BP transmission in our cohort (76% or 2,219 of 2920 had no BP transmission), we dichotomized this measure, using the “any vs. none” definition as opposed to more explanatory definitions owing to data skewness and zero inflation. No BP transmission was coded as “no transmission” and ≥ 1 transmission coded as “high BP transmission.”

Clinician interactions were defined as the frequency of clinician interactions using video and telephone visits and secure messaging within the study period. This measure did not include in-person visits, as we aimed to measure digitally enabled RPM interactions with clinicians. Encounter data for each interaction point was collected and stored using an activity-log system within NYULH’s larger Epic ecosystem and later transmitted and stored in NYULH’s data warehouse. We dichotomized this measure at the median number of interaction events, which were 2 interaction events (< 2 interactions coded as low clinician interaction vs. ≥ 2 interactions coded as high clinician interaction).

Patient portal interactions were defined as the frequency of logins to NYU’s MyChart patient portal account. Patient portal interaction data were extracted from recorded time-stamped frequency logs, which are routinely captured by Epic and stored in NYULH’s data warehouse. This measure was dichotomized at the median threshold, or 22 portal interaction events (< 22 interactions coded as low portal interactions: Quartile 1 and 2 vs. ≥ 22 interactions coded as high portal interactions: Quartile 3 and 4).

### Covariates

Based on our a priori assessments, including our review of the available literature, [28] we determined that the following covariates were potential confounders for the relationship between our exposure and outcome variables: age, sex, insurance status, smoking status, office visits, number of anti-hypertensive medications, comorbidities (obesity, type 2 diabetes, chronic kidney disease, hyperlipidemia) and clinical practice types. Age was operationalized as a continuous variable in our statistical models, but was categorized in Table 2 for illustrative purposes across the following categories: 18–24, 25–29, 40–54, 55–64, > 65. Since we anticipate that participants with advanced stages of HTN may be higher utilizers of RPM, we adjusted for baseline severity of HTN across 4 stages: normal (<120/80 mm Hg), elevated (120–129 mm Hg/ < 80 mm Hg), stage 1 high BP (130–139 mm Hg/ 80–89 mm Hg), stage 2 and beyond high BP (≥ 140/ ≥ 90 mm Hg). [40] Further, to isolate the direct influence of each exposure measure, we controlled for two of the three utilization measures in each statistical model. Our study period spanned January 1, 2018, to January 1, 2024, reflecting a 6-year observation period. To assess the influence of the pandemic on BP outcomes, we conducted a priori analyses across two distinct periods: January 1st, 2018, to March 23rd, 2020, vs March 24th, 2020, and onwards, reflecting our pre- vs post-pandemic assessment periods. January 1, 2018, to March 5th, 2023, vs March 6, 2023, to January 1, 2024, represented our pre-/during- vs post-pandemic periods, with March 23, 2023, serving as the end date for the pandemic period as declared by the World Health Organization [33]. Periods that were marked by statistically significant differences in BP outcomes were controlled for in our statistical models.

**Table 2:** Frequency and percentage of sociodemographic characteristics, BP outcomes, and RPM utilization by race/ethnicity, with corresponding *p*-values from Chi-squared tests.

Demographics	Total	Non-Hispanic White	Non-Hispanic Black	Hispanic	Other/unknown	P-value
	2920 (100)	693 (24)	776 (27)	1142 (39)	309 (11)	
**Sex**						< 0.001
Male	1197 (41)	314 (45)	229 (30)	514 (45)	140 (45)	
Female	1721 (59)	378 (55)	547 (70)	628 (55)	169 (54)	
**Age**						< 0.001
18-24	19 (1)	5 (0.72)	6 (0.80)	6 (0.52)	2 (0.64)	
25-29	321 (11)	70 (10)	99 (12)	100 (9)	52 (17)	
40-54	767 (26)	121 (17.5)	232 (30)	341 (30)	73 (23)	
55-64	721 (25)	162 (23.4)	221 (28.5)	273 (24)	65 (21)	
> 65	1092 (37)	335 (48)	218 (28)	422 (37)	117 (38)	
**Insurance status**						** **< 0.001
Private	1027 (35)	315 (45)	327 (42)	229 (20)	156 (50)	
Public	1593 (55)	293 (42)	403 (52)	774 (68)	123 (40)	
Other	300 (10)	85 (12)	46 (6)	139 (12)	30 (10)	
**BP Categories**						< 0.001
Normal	109 (4)	44 (6)	19 (2)	24 (0.08)	22 (7)	
Elevated	79 (3)	46 (7)	10 (1)	12 (1)	11 (4)	
Stage 1	1258 (43)	322 (46)	302 (39)	491 (43)	143 (46)	
Stage 2 +	1452 (50)	273 (39)	443 (57)	610 (53)	126 (41)	
Unknown	22 (0.8)	8 (1)	2 (0.26)	5 (0.44)	7 (2)	
**BP control (JNC-8 definition of control BP 140/90)**						0.001
Controlled	2258 (77)	557 (80)	583 (75)	904 (79)	214 (69)	
Uncontrolled	662 (23)	136 (20)	193 (25)	238 (21)	95 (31)	
**Frequency and percentage of RPM utilization by race**						** **< 0.001
High BP transmission (≥1)	714 (24)	280 (40)	166 (21)	173 (15)	95 (31)	
High clinician interactions (≥2)	1235 (42)	424 (61)	362 (47)	306 (27)	143 (47)	
High patient portal interactions (≥22)	1440 (49)	464 (67)	384 (49)	422 (37)	170 (55)	

Chi-squared tests were used to compare categorical variables across race/ethnicity

### Multicollinearity assessments

We conducted pairwise correlation and generalized variance inflation factors (GVIF) assessments to evaluate potential multicollinearity. We utilized coefficients from GVIF analyses in logistic regression models, which included the main effects from our utilization indicators, race/ethnicity, and all adjustment covariates. In addition, we assessed the internal consistency of each utilization domain by calculating Cronbach’s alpha coefficients to ascertain the reliability of our RPM utilization indicators.

### Data analysis

We conducted our analyses using R version 4.4.3 [34]. Descriptive analyses were performed to characterize participants’ demographic characteristics. Frequencies and percentages were computed using Chi-squared tests, which are reported with p-values. (**Table 2**) We used logistic regression (i.e., specifying the family as binomial and using the logit link), to estimate the adjusted and unadjusted relationships for two-way interactions between race and RPM utilization intensity in their effects on BP control, using JNC-8- and ACC/AHA-defined outcome variables. The race/ethnicity categories include Hispanics, non-Hispanic Whites, non-Hispanic Blacks, and Other/unknown. Due to small sample sizes, the following groups were collapsed into the Other/unknown category: Asians, American Indians and Alaska Natives (NI/AN), Native Hawaiians and Pacific Islanders (NH/PI), and unknown observations. Multivariable logistic regression models were used for covariate adjustments.

We also examined adjusted odds ratios (OR) for uncontrolled BP by race/ethnicity and utilization group combination, with high-utilization non-Hispanic White as the reference category. Each model compared combinations of race/ethnicity and our healthcare utilization measures against the reference group of the high-utilization non-Hispanic White combination. The adjusted odds ratios, 95% confidence intervals (CI), and p-values are shown for both the primary and secondary outcomes.

### Sensitivity analysis

To support our primary analyses, we conducted a principal component analysis (PCA) to derive a composite engagement score from the three utilization variables. This score was then included in logistic regression models adjusted for demographics, comorbidities, and baseline BP, and tested for interaction with race/ethnicity.

### Ethics approval and data accession

This study was approved by the New York University Institutional Review Board, which waived requirements for informed consent for analysis of de-identified data. Data for this work was accessed on May 17, 2024. Full access to patient data was granted only to our lab’s data analyst and the first author (JMM) until the data were de-identified. All data were stored on a secure, institution-provided computer or laptop.

## Results

**Fig 1:**
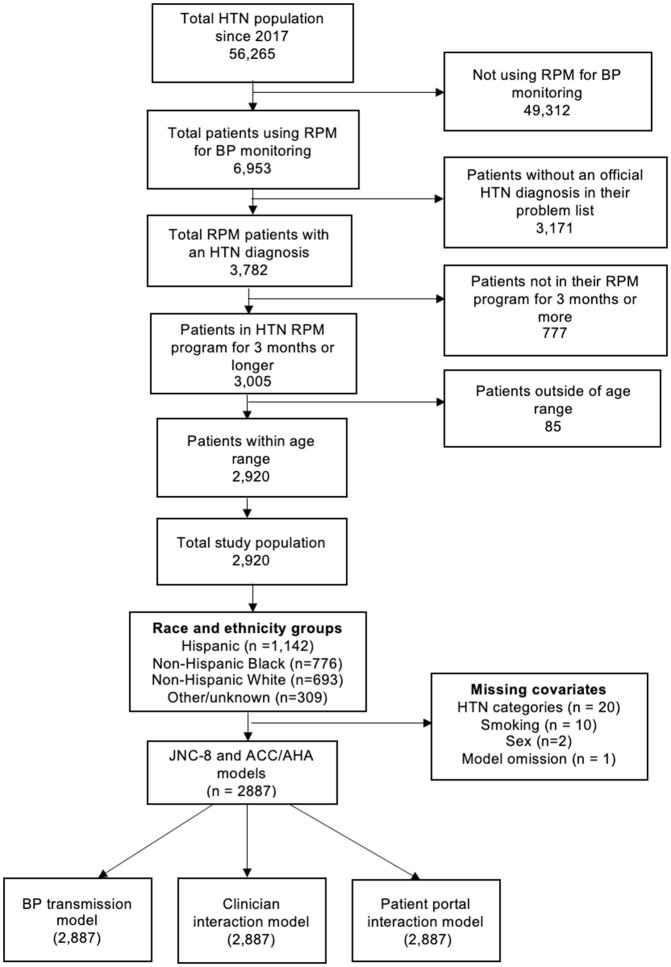
CONSORT diagram of study sample inclusion.

Of 2920 participants (**Fig 1**) in the analytical sample, 721 (59%) were females. Seven hundred and sixty-seven (26%) were between the ages of 40 and 54 years old and 721 (25%) were between the ages of 55 and 64 years old. Among race/ethnicity groups, 1142 (39%) were Hispanic, 776 (27%) were non-Hispanic Black, 693 (24%) were non-Hispanic White and 309 (11%) patients were categorized as Other/unknown. Private insurance coverage was highest among non-Hispanic White patients, 315 (45%), followed by non-Hispanic Black and Hispanic patients, 327 (42%) and 229 (20%), respectively, while public insurance was highest among Hispanic patients 774 (68%), followed by non-Hispanic Black and non-Hispanic White patients 403 (52%) and 293 (42%), respectively.

### A-priori assessments of pandemic influence on BP outcomes

Our a-priori analyses demonstrated no statistically significant differences in BP outcomes between our pre- and post-pandemic periods: unadjusted OR 0.95 (95% CI: 0.72–1.24) and adjusted OR 0.99 (95% CI: 0.73–1.33). However, there were statistically significant differences between our pre-/during-pandemic and post-pandemic cohorts. These differences were adjusted for in our analytical models: Unadjusted OR 3.33 (95% CI: 2.71–4.09), and adjusted OR 4.13 (95% CI: 3.30–5.18).

### Correlational analyses

Pairwise correlations revealed moderate associations among the three RPM utilization variables: clinician interactions and patient portal interactions (r = 0.54); BP transmission and patient portal interactions (r = 0.43), and BP transmission and clinician interactions (r = 0.33). GVIF values were all below 2 across exposures and covariates, indicating no concerning multicollinearity. Additionally, Cronbach’s alpha for the three utilization measures was 0.697, reflecting moderate internal consistency and interrelatedness without too much redundancy.

### Prevalence of RPM utilization by race and ethnicity (Table 2)

Among the study sample, only 714 (24%) submitted BP measurements throughout the study period. Chi-squared tests showed that non-Hispanic White patients had the highest proportion of BP transmission (p < 0.001): 280 (40%, 95% CI: 37–44), followed by non-Hispanic Black and Hispanic patients: 166 (21%, 95% CI: 19–24%) and 173 (15%, 95% CI: 13–17%), respectively. Similar trends in utilization were observed for clinician interactions, where non-Hispanic White patients had 424 (61%, 95% CI: 57–65%) clinician engagements, compared to non-Hispanic Black and Hispanic patients at 362 (47%, 95% CI: 43–50%) and 306 (27%, 95% CI: 24–27%), respectively. Non-Hispanic Whites similarly had the highest patient portal interactions, 464 (67%, 95% CI: 63–70%), followed by non-Hispanic Black patients and Hispanic patients, 384 (49%, 95% CI: 46–53%) and 422 (37%, 95% CI: 34–40%), respectively.

### Effect modification assessments of RPM utilization and race/ethnicity, using JNC-8 primary outcome (BP > 140/90)

We tested for effect modification across strata of race/ethnicity. Significant interaction effects were observed for Hispanic patients in two domains. Specifically, the interaction between high BP submission and Hispanic ethnicity was statistically significant (interaction OR = 2.00, p = 0.029), indicating that the association between high BP transmission and BP control was twice as strong for Hispanic patients compared to non-Hispanic White patients. Similarly, the interaction between high portal interaction and Hispanic ethnicity was also significant (interaction OR = 1.98, p = 0.010), suggesting that the association of high patient portal engagement on BP control was more pronounced among Hispanic patients. In contrast, no statistically significant interactions were observed for clinician interaction or for any other racial/ethnic group (all p-values > 0.13). These results suggest that the relationship between RPM engagement and BP control may vary by race/ethnicity, particularly among Hispanic patients.

**Table 3** provides both unadjusted and adjusted race-specific associations between RPM utilization and uncontrolled BP (the primary outcome). For BP transmission, “no BP transmission” was associated with significantly increased odds of uncontrolled BP (OR = 1.71, p = 0.025) among non-Hispanic White and (OR = 1.93, p = 0.013) among non-Hispanic Black race, respectively, compared to those with high BP transmission of the same race/ethnicity group. We observed statistically significant associations between clinician interaction and uncontrolled BP across all race/ethnicity groups: (OR = 2.37, p = 0.00) for non-Hispanic White, (OR = 2.75, p = 0.00) for non-Hispanic Black, (OR = 1.61, p = 0.020) for Hispanic, and (OR = 2.90, p = 0.000) for patients categorized as “Other,” respectively, compared to those with high clinician interactions of the same race/ethnicity group. Similar findings were observed for portal interactions, except among Hispanic ethnicity.

**Table 3:** Adjusted and unadjusted associations between RPM utilization intensity and blood pressure (BP) control (uncontrolled vs. controlled), stratified by race (JNC-8 definition of blood pressure control 140/90). This table presents the prevalence of uncontrolled BP and corresponding odds ratios (ORs) for each RPM utilization measure across racial/ethnic groups, using the JNC-8 definition of BP control. Multivariable logistic regression models adjusted for age, sex, insurance, smoking status, comorbidities, clinical practice type, baseline BP, number of medications, number of office visits, pandemic timing, and the remaining two RPM utilization indicators. High RPM utilization is the reference category for each domain. BP control is the referent outcome.

RPM utilization measures	Non-Hispanic^f^ Whites				Non-Hispanic Blacks				Hispanics				Other/unknown			
	n^a^	% uncon. BP^b^	Unadj.OR (95% CI,p-value)	Adjusted OR (95% CI, p-value)	n^a^	% uncon.BP^b^	Unadj.OR (95% CI, p-value)	Adjusted OR (95% CI, p-value)	n^a^	% uncon.BP^b^	Unadj. OR (95% CI, p-value)	Adjusted OR (95% CI, p-value)	n^a^	% uncon.BP^b^	Unadj.OR (95% CI, p-value)	Adjusted OR (95% CI, p-value)
**High BP**^**c**^ **transmission** (≥ 1)	280	15	Ref.	Ref.	166	17	Ref.	Ref.	173	24	Ref.	Ref.	95	25	Ref.	Ref.
**No BP transmission**	413	32	2.07 (1.37-3.13, 0.0006)	1.71 (1.07-2.75, 0.025)	610	37	1.83 (1.17-2.85, 0.0077)	1.93 (1.15-3.23, 0.013)	951	27	1.14 (0.76-1.71, 0.535)	0.89 (0.56 −1.43, 0.641)	214	55	2.20 (1.24-3.92, 0.0071)	1.37 (0.71-2.66, 0.353)
**High**^**d**^ **clinician interactions** (≥2)	424	14	Ref.	Ref.	362	15	Ref.	Ref.	306	17	Ref.	Ref.	143	16	Ref.	Ref.
**Low clinician** **interactions** (<2)	269	31	3.12 (2.12-4.60, < .0001)	2.37 (1.52-3.70, 0.000)	414	34	3.08 (2.15 - 4.40, < .0001)	2.75 (1.84-4.11, < .000)	836	23	1.86 (1.30-2.67, 0.000)	1.61 (1.08-2.40, 0.020)	166	43	4.00 (2.33-6.87, < .0001)	2.90 (1.56-5.42, 0.000)
**High patient portal**^**e**^ **interactions** (≥22)	464	13	Ref.	Ref.	384	15	Ref.	Ref.	422	18	Ref.	Ref.	170	19	Ref.	Ref.
**Low patient portal interactions** (<22)	229	33	3.22 (2.19-4.73, < .0001)	2.07 (1.37-3.13, 0.00)	392	34	2.95 (2.08-4.18, < .0001)	1.83 (1.17-2.85, 0.008)	720	23	1.39 (1.02-1.88, 0.036)	1.14 (0.56-1.71, 0.535)	139	45	3.57 (2.15-5.95, < .0001)	2.20 (1.24-3.92, 0.007)

^a^n refers to the number of participants in each utilization category.

^b^% uncon. refers to percentage of uncontrolled BP for each utilization category.

^c^BP outcomes were dichotomized as controlled vs. uncontrolled BP based on the JNC-8 definition of control: SBP >140 mm Hg / DBP <90 mm Hg.

^d^High utilization is the referent group for each exposure measure: BP transmission (no BP transmission vs. ≥ 1 transmissions); Clinician interactions were dichotomized as low (<2 interactions) or high (≥2 interactions), corresponding approximately to the lower (Q1–Q2) and upper (Q3–Q4) halves of the distribution, respectively.

^e^Patient portal use was categorized as low (<22 interactions; Q1–Q2) vs. high (≥22 interactions; Q3–Q4).

^f^non-Hispanic White patients are the reference group for the sample.

**Table 4** reports unadjusted and adjusted associations between RPM utilization and uncontrolled BP across race/ethnicity, using our ACC/AHA outcome definition. For BP transmission, adjusted analyses demonstrated statistically significant associations between no BP transmission and uncontrolled BP across all racial groups (except Hispanic ethnicity): (OR = 1.84, p = 0.002) for non-Hispanic White, (OR = 1.55, p = 0.036) for non-Hispanic Black, and (OR = 2.03, p = 0.020) for “Other” racial groups, respectively, compared to those with high BP transmission at a stricter level of BP control of the same racial group. Similar and statistically meaningful associations between clinician interactions and uncontrolled BP were observed among all race/ethnicity groups: (OR = 1.72, p = 0.004) for non-Hispanic White, (OR = 2.11, p = < .0001) for non-Hispanic Black, (OR = 1.68, p = 0.001) for Hispanic, and (OR = 2.84, p = 0.000) for “Other” racial group, respectively. Similar patterns were observed for portal interactions among non-Hispanic White, Hispanic and “Other” race/ethnicity groups, but not among non-Hispanic Black patients.

**Table 4:** Adjusted and unadjusted associations between RPM utilization intensity and blood pressure (BP) control (uncontrolled vs. controlled), stratified by race (ACC/AHA definition of blood pressure control 130/90). This table presents the prevalence of uncontrolled BP and corresponding odds ratios (ORs) for each RPM utilization measure across racial/ethnic groups, using the ACC/AHA definition of BP control. Multivariable logistic regression models adjusted for age, sex, insurance, smoking status, comorbidities, clinical practice type, baseline BP, number of medications, number of office visits, pandemic timing, and the remaining two RPM utilization indicators. High RPM utilization is the reference category for each domain. BP control is the referent outcome.

RPM utilization measures	Non-Hispanic ^f^ Whites				Non-Hispanic Blacks				Hispanics				Other/unknown			
	n^a^	% uncon.BP^b^	Unadj. OR (95% CI, p-value)	Adjusted OR (95% CI, p-value)	n^a^	% uncon. BP^b^	Unadj. OR (95% CI, p-value)	Adjusted OR (95% CI, p-value)	n^a^	% uncon. BP ^b^	Unadj. OR (95% CI, p-value)	Adjusted OR (95% CI, p-value)	n^a^	% uncon. BP^b^	Unadj. OR (95% CI, p-value)	Adjusted OR (95% CI, p-value)
**High BP** ^**c**^ **transmission** (≥ 1)	280	30	Ref.	Ref.	166	34	Ref.	Ref.	173	36	Ref.	Ref.	95	32	Ref.	Ref.
**No BP transmission**	413	65	2.16 (1.53-3.03, < .000)	1.84 (1.26-2.70, 0.002)	610	46	1.61 (1.13-2.31, 0.009)	1.55 (1.03-2.35, 0.036)	951	43	1.30 (0.93-1.82, 0.124)	1.09 (0.74 −1.59, 0.670)	214	56	2.77 (1.66-4.61, 0.000)	2.03 (1.14-3.63, 0.020)
**High** ^**d**^ **clinician interactions** (≥2)	424	25	Ref	Ref.	362	32	Ref.	Ref.	306	30	Ref.	Ref.	143	31	Ref.	Ref.
**Low clinician** **interactions** (<2)	269	45	2.42 (1.75-3.35, < .0001)	1.72 (1.19-2.50, 0.004)	414	53	2.46 (1.83-3.30, < .0001)	2.11 (1.52-2.93, < .0001)	836	45	1.96 (1.48-2.59, < .0001)	1.68 (1.23-2.30, 0.001)	166	64	3.98 (2.47-6.40, < .0001)	2.84 (1.66-4.48, 0.000)
**High patient portal** ^**e**^ **interactions** (≥22)	464	26	Ref.	Ref.	384	34	Ref.	Ref.	422	34	Ref.	Ref.	170	34	Ref.	Ref.
**Low patient portal interactions** (<22)	229	46	2.49 (1.78-3.47, < .0001)	1.87 (1.36-2.56, 0.000)1	392	52	2.12 (1.59-2.83, < .0001)	1.11 (0.862-1.43, 0.414)	720	46	1.65 (1.29-2.12, 0.000)	1.73 (1.36-2.20, < .0001)	139	66	3.78 (2.35-6.07, < .0001)	2.88 (1.83-4.52, < .0001)

^a^n refers to the number of participants in each utilization category.

^b^% uncon. refers to percentage of uncontrolled BP for each utilization category.

^c^BP outcomes were dichotomized as controlled vs. uncontrolled BP based on the JNC-8 definition of control: SBP >140 mm Hg / DBP <90 mm Hg.

^d^High utilization is the referent group for each exposure measure.

^e^Patient portal use was categorized as low (<22 interactions; Q1–Q2) vs. high (≥22 interactions; Q3–Q4).

^f^non-Hispanic White patients are the reference group for the sample.

In **Table 5**, we report adjusted odds ratios for uncontrolled BP by race/ethnicity and utilization group combination, with high-utilization non-Hispanic White as the reference category for both outcomes. Each model compares combinations of race/ethnicity and healthcare utilization against the reference group of high-utilization non-Hispanic White patients. We observed significantly higher odds of uncontrolled BP among patients from other racial/ethnic groups with low engagement across all three utilization domains. For example, in **Table 5**, among patients with low clinician interaction, the odds of uncontrolled BP were over three times higher for non-Hispanic Black patients (adjusted OR = 3.08, 95% CI: 1.99–2.78) and more than 3.6 times higher for patients categorized as “Other” (adjusted OR = 3.69, 95% CI: 2.27–6.01) compared to the reference group. Similar patterns were observed for low patient portal interaction, particularly among non-Hispanic Black and “Other” racial groups. These disparities remained consistent when using a stricter outcome definition in sensitivity analyses (secondary outcome).

**Table 5:** RPM utilization combinations with race/ethnicity for primary and secondary outcomes, using the non-Hispanic Whites and high utilization combination as the reference group, for each of the three domains: BP transmission, Clinician interaction, patient portal interaction.

		Primary outcome: JNC-8 definition BP > 140/90			Secondary outcome: ACC/AHA definition BP > 130/80		
	Race-utilization combination	Adjusted Odds Ratio	95% CI	P-value	Adjusted Odds Ratio	95% CI	P-value
	The reference group: White-High utilization (separately for each domain: BP transmission, Clinician interaction, patient portal interaction)						
**BP transmission** (No transmission vs. High:** **≥ 1 transmission)	White-No transmission	1.72	(1.07-2.75)	0.024*	1.84	(1.25-2.70)	0.002*
	Black-No transmission	2.11	(1.32-3.39)	0.002*	2.01	(1.37-2.95)	0.002*
	Hispanic-No transmission	1.05	(0.65-1.67)	0.853	1.36	(0.93-1.98)	0.109
	Other-No transmission	2.36	(1.41-3.96)	0.001*	2.82	(1.82-4.37)	<.0001*
	Black-High	1.10	(0.65-1.84)	0.771	1.30	(0.80-2.10)	0.291
	Hispanic-High	1.17	(0.65-2.9)	0.599	1.25	(0.78-2.00)	0.348
	Other-High	1.72	(0.87-3.43)	0.122	1.39	(0.78-2.46)	0.265
**Clinician interaction** (Low: < 2 interactions vs. High: ≥ 2 interactions)	White-Low	2.37	(1.52-3.70)	0.000*	1.72	(1.19-2.50)	0.004*
	Black-Low	3.08	(1.99-4.78)	<.0001*	2.22	(1.55-3.19)	<.0001*
	Hispanic-Low	1.50	(0.99-2.28)	0.053	1.47	(1.06-2.04)	0.020*
	Other-Low	3.69	(2.27-6.01)	<.0001*	3.26	(2.11-5.03)	<.0001*
	Black-High	1.12	(0.70-1.79)	0.631	1.05	(0.74-1.51)	0.773
	Hispanic-High	0.93	(0.58-1.52)	0.783	0.88	(0.60-1.27)	0.487
	Other-High	1.27	(0.70-2.30)	0.426	1.15	(0.72-1.82)	0.558
**Patient portal interaction** (Low < 22 interactions vs. High: ≥ 22 interactions)	White-Low	2.27	(1.45-3.57)	0.000*	1.79	(1.12-2.63)	0.003*
	Black-Low	2.93	(1.91-4.50)	<.0001*	2.06	(1.44-2.94)	<.0001*
	Hispanic-Low	1.23	(0.81-1.86)	0.337	1.37	(0.98-1.91)	0.065
	Other-Low	3.24	(1.96-5.16)	<.0001*	4.26	(2.67-6.78)	**<.0001**
	Black-High	1.10	(0.70-1.71)	0.685	1.17	(0.83-1.64)	0.367
	Hispanic-High	1.06	(0.69-1.62)	0.786	0.93	(0.67-1.31)	0.709
	Other-High	1.48	(0.88-2.49)	0.078	1.31	(0.86-1.99)	0.202

### Sensitivity analyses

We conducted a sensitivity analysis using a composite RPM engagement score based on the first principal component (PC1). The findings were consistent with our main results: Lower PC1 scores—indicating lower overall engagement—were significantly associated with higher odds of uncontrolled BP (OR = 1.77; 95% CI: 1.52–2.10; *p* < 0.001). We also observed a significant interaction between PC1 and race/ethnicity (*p* = 0.006), indicating that the protective association between RPM engagement and BP control was strongest among non-Hispanic White patients and attenuated among Hispanic patients.

## Discussion

The present study sought to descriptively characterize disparities in BP outcomes among a diverse sample of NYULH hypertensive patients and assess the presence of effect modification by race in the relationship between RPM utilization intensity and uncontrolled BP. Our findings illustrate existing disparities in utilization between non-Hispanic Whites and non-Hispanic Black, Hispanic and “Other” race/ethnicity groups across measures of RPM utilization. We observed differences in uncontrolled BP prevalence, with non-Hispanic White patients having the lowest prevalence of uncontrolled BP (20%) relative to Hispanic (21%) and non-Hispanic Black patients (25%). We observed statistically meaningful effect modification according to race for the associations between low RPM utilization intensity and uncontrolled BP. Particularly, our findings suggest that, when compared to other groups, Hispanic patients are least poised to benefit from increased engagement with RPM utilization metrics. Additionally, when facilitating direct comparisons with a common reference group, high-utilization non-Hispanic Whites, significantly increased odds of uncontrolled BP was observed for non-Hispanic Black and “Other” racial groups.

Our findings of increased RPM utilization among non-Hispanic White patients across all utilization measures are consistent with existing studies demonstrating disparities in telemedicine utilization across race/ethnicity groups. A preponderance of scientific evidence emerging from the recent pandemic has shown that non-Hispanic White patients utilize telemedicine in greater proportions relative to racial/ethnic minorities, though this is often context-specific [35–38]. Furthermore, our findings support the notion that disparities in utilization may potentially be linked to BP outcomes, as patients with lower utilization across all representative groups had a higher prevalence of uncontrolled BP. These findings lend credence to the resounding claims that the expansion of telemedicine exacerbated existing health inequities during the pandemic [39,40]. While research is still scarce on the particular drivers of inequities in BP transmission, for instance, there is available research highlighting disparities in patient-clinician interactions, which are associated with worse HTN and cardiovascular outcomes, including medication adherence and atherosclerosis among racial/ethnic minorities [41–46]. Disparities in patient portal use are well documented and have been widely associated with the term “intervention-generated inequalities” (IGI) in informatics research [47–49]. IGI relates to inequities that arise when digital interventions are designed to benefit segments of the population that are more advantaged and predisposed to using those interventions [50].

While the prevalence of uncontrolled BP in our sample was below nationally representative estimates, [1] our findings on disparities in HTN control are still microcosmic of existing disparities in HTN outcomes. There is a wide body of research showcasing that non-Hispanic White patients often experience better BP control rates relative to racial/ethnic minorities [5,51–55]. Furthermore, our study showed that non-Hispanic Black patients had the highest rates of uncontrolled BP relative to other groups, which is widely documented in the HTN disparities literature [5,51,53,56]. Hispanic patients had a near parity prevalence of uncontrolled BP relative to non-Hispanic Whites while having lower utilization compared to all other groups. This is consistent with available literature showing that Hispanic patients utilize telemedicine at lower rates while simultaneously experiencing HTN outcomes at parity or better than non-Hispanic White patients [53,57–60]. However, utilization patterns among racial/ethnic populations are not uniform across all contexts and patient populations [57,61]. The paradoxical findings observed among Hispanic patients are commonly referred to as the “Hispanic Health Paradox,“ whereby Hispanic patients are reported to have better than expected health outcomes despite experiencing lower average SES and other impediments to healthcare, including language and digital access barriers [59,62]. Efforts aimed at reducing health and utilization disparities among this population should center on transforming our current healthcare delivery system into a more digitally, culturally, and linguistically responsive system.

Our models conducting direct comparison between racial/ethnic groups, using high-utilization non-Hispanic White patients as the reference group, demonstrated increased likelihood of uncontrolled BP for racial/ethnic groups with low RPM utilization relative to their high-utilization non-Hispanic White counterparts. These findings unveiled disparities in RPM that mirror underlying inequities within the broader healthcare delivery system. Observed lower RPM utilization among racial/ethnic groups and disproportionately elevated odds of uncontrolled BP among low utilizing non-Hispanic Black and “Other” racial groups, consisting of Asians, AI/NA, NH/PI, illustrate the potential consequences underlying low participatory engagement in RPM programs for minority populations in a rapidly evolving digital health landscape [26,62,63]. Furthermore, our results support existing digital health/inequity research, highlighting the benefits of digital health technologies and advocating the need to address digital inequities with urgency, particularly since we observed no difference in the likelihood of being uncontrolled when comparing racial/ethnic groups with high RPM utilization to their high-utilization non-Hispanic White counterparts. [19,64–67] Given these findings, further investigation is warranted on the independent associations between heightened technology use in the post-pandemic era and clinical outcomes, generally, and across various sociodemographic subgroups.

### Limitation

Findings from this study should be interpreted in the context of several limitations. First, this study examined both self-measured and clinic-recorded BP measurements for our outcome variables. While home-measured BP is more accurate than clinic-recorded BP, [68,69] errors occurring during BP ascertainment could have induced measurement bias in the study. Second, challenges in procuring a digital device could have limited participation among lower-income individuals who may not be able to afford an iPhone, which is often more compatible with the RPM program components than Android devices. While we controlled for health insurance as a proxy for SES, patients who struggled to acquire digital devices needed for the program may have been precluded from participation. As such, our sample may be limited to participants who are more socio-economically advantaged or more inclined to use digital technologies to manage their health. Fourth, we used a complete sample of urban-dwelling hypertensive patients from NYULH. Findings, therefore, cannot be generalized to other patient populations or geographic contexts. Fifth, since OR inflates the risk ratio for disease prevalence above 10%, the OR estimates observed in our findings are likely inflated in comparison to the risk estimates [70]. Sixth, a wide range of confounders was controlled for in our study. However, other possible confounders were not adjusted for, such as medication adherence and social determinants of health factors, including social and physical environments [71] and digital infrastructure, [72] as these data are seldom captured in the EHR. This may have induced unmeasured confounding in our study.

## Conclusion

As RPM programs become increasingly ubiquitous and telemedicine use becomes a routinized part of HTN care processes, understanding differential use patterns among vulnerable groups, including racial/ethnic minority populations, will become more salient in the times to come. Our study provides early evidence of differences in uncontrolled BP prevalence and utilization patterns according to race/ethnicity, and the presence of effect modification by race in the relationship between RPM utilization intensity and uncontrolled BP among our urban-dwelling HTN patient population. Future research programs should seek to understand how utilization trends among various vulnerable population groups, including racial/ethnic minorities, influence HTN outcomes.

## Supporting information

S1 File RPM utilization and Uncontrolled HTN: Variations by race study dataset. (CSV)

S2 File RPM utilization and Uncontrolled HTN: Variations by race study codebook. (DOCX)
