# Supplementary material for: Associations between remote patient monitoring and uncontrolled blood pressure among patients diagnosed with hypertension: Exploring variations by race/ethnicity
Source: PLoS One. 2025 Nov 6;20(11):e0334887. doi: 10.1371/journal.pone.0334887 (PMC12591402; doi:10.1371/journal.pone.0334887)
Supplement: S2 File — (DOCX) [file pone.0334887.s002.docx]

**RPM Utilization and Uncontrolled HTN: Variations by race study codebook**

**Variables: Outcome measures**

| **Outcome**  **measure** | **Variable name** | **Code** | **Definition** |
| --- | --- | --- | --- |
| Primary outcome  (BP <140/90) | Nqoutcome1 | Dichotomized  (0 = controlled, 1= uncontrolled) | Controlled BP = BP < 140/90  Uncontrolled BP = <130/80 |
| Secondary outcome  (BP <130/80) | Ahaoutcome1 | Dichotomized  (0 = controlled, 1= uncontrolled) | Controlled BP = BP < 130/80  Uncontrolled BP = >130/80 |

s

**Variables: Exposure measures**

| **Exposure measure** | **Variable name** | **Code** | **Definition** |
| --- | --- | --- | --- |
| BP submission | nbpsub | 0 = High utilization, 1 = low utilization | High utilization = more than 1 BP submission. Low utilization = no submission |
| Clinician interaction | dcatpi | 0 = High utilization, 1 = Low utilization | High utilization = more than 2 clinician interactions. Low utilization = less than 2 clinician interactions. |
| Patient portal interaction | dcatpaccess | 0 = High utilization, 1 = Low utilization | High utilization = more than 22 portal interactions. Low utilization = less than 22 interactions |

**Covariates**

| **Exposure measure** | **Variable name** | **Code** | **Definition** |
| --- | --- | --- | --- |
| Race | nrace | 4 levels: 0-3 | 0 = NH White  1 = Nh Black  2 = Hispanic  3 = Other |
| Age | ageinyears | Continuous | Participant age in years |
| Smoking | smoking | 3 levels:  0-2 | 0 = Nonsmoker  1 =Smoker  2 = Unknown |
| Insurance | insurance | 3 levels:  0-2 | 0 = Private  1 = Public  2 = Other |
| Hypertension categories  (baseline) | htncatz | 5 levels:  0-4 | 0 = Normal  1 = Elevated  2 = Stage 1  3 = Stage 2  4 = Unknown |
| Department speciality | department_speciality | 11 levels:  0-10 | A range of departments participating in remote patient monitoring across the enterprise |
| Comorbidities:  Type 2 diabetes  Chronic kidney disease  Hyperlipidemia  Obesity | type.2.diabetes  chronic.kidney.disase  hyperlipidemia  obesity | 2 levels:  0-1 | Hypertension comorbidities  0 = No  1 =Yes |
| Office visits | ovisits | 2 levels:  0-1 | Defined based on whether the patient had an office visit during the 3-month observation period    0 = No  1 = Yes |
| Number of medications | medications | Continuous | The number of medications taken by patients during the observation period |
| Pre- vs during/post- pandemic | covidera | 2 levels:  0-1 | Pre- vs pandemic/post-pandemic participants  0 = pre-pandemic participants  1 = pandemic era participants |
| Pre-/during- vs post-pandemic | postpandemic | 2 levels:  0-1 | Pre-/during-pandemic vs post-pandemic participants  0 = pre-/during-pandemic participants  1 = post-pandemic era participants |
